# Proteomic patterns analysis with multivariate calculations as a promising tool for prompt differentiation of early stage lung tissue with cancer and unchanged tissue material

**DOI:** 10.1186/1746-1596-6-22

**Published:** 2011-03-21

**Authors:** Piotr Waloszczyk, Tomasz Janus, Jacek Alchimowicz, Tomasz Grodzki, Krzysztof Borowiak

**Affiliations:** 1 Department of Toxicology and Molecular Pathobiochemistry, Pomeranian Medical University, 70-204 Szczecin, Poland; 2 Professor A. Sokolowski Specialist Hospital Szczecin Zdunowo, Poland

## Abstract

**Background:**

Lung cancer diagnosis in tissue material with commonly used histological techniques is sometimes inconvenient and in a number of cases leads to ambiguous conclusions. Frequently advanced immunostaining techniques have to be employed, yet they are both time consuming and limited. In this study a proteomic approach is presented which may help provide unambiguous pathologic diagnosis of tissue material.

**Methods:**

Lung tissue material found to be pathologically changed was prepared to isolate proteome with fast and non selective procedure. Isolated peptides and proteins in ranging from 3.5 to 20 kDa were analysed directly using high resolution mass spectrometer (MALDI-TOF/TOF) with sinapic acid as a matrix. Recorded complex spectra of a single run were then analyzed with multivariate statistical analysis algorithms (principle component analysis, classification methods). In the applied protocol we focused on obtaining the spectra richest in protein signals constituting a pattern of change within the sample containing detailed information about its protein composition. Advanced statistical methods were to indicate differences between examined groups.

**Results:**

Obtained results indicate changes in proteome profiles of changed tissues in comparison to physiologically unchanged material (control group) which were reflected in the result of principle component analysis (PCA). Points representing spectra of control group were located in different areas of multidimensional space and were less diffused in comparison to cancer tissues. Three different classification algorithms showed recognition capability of 100% regarding classification of examined material into an appropriate group.

**Conclusion:**

The application of the presented protocol and method enabled finding pathological changes in tissue material regardless of localization and size of abnormalities in the sample volume. Proteomic profile as a complex, rich in signals spectrum of proteins can be expressed as a single point in multidimensional space and than analysed using advanced statistical methods. This approach seems to provide more precise information about a pathology and may be considered in futer evaluation of biomarkers for clinical applications in different pathology. Multiparameter statistical methods may be helpful in elucidation of newly expressed sensitive biomarkers defined as many factors "in one point".

## Background

Cancer diagnosis based on standard histological methods is widely described and used in medicine. However, most of the procedures derive from a subjective assessment of observed changes and in some cases may be inconclusive. In practical terms, new, objective methods in cancer diagnosis are still needed especially in respect of their sensitive. Moreover, tumour growth in its early stages is restricted to only a part of the tissue and in some cases it may be overlooked. Recently, proteomics has proved to be a valuable approach in biomarker detection and finding sensitive parameters which indicate disease process [1-4]. The combination of spectroscopic methods of high resolution (mass spectroscopy, nuclear magnetic resonance) with advanced statistical methods leads to an increased likelihood of developing new applications for diagnostic purposes [5-9]. Molecular imaging techniques described in literature as well as profiling and detailed characterisation of biomarker methods tend to prove their usefulness [10-12]. However, many protocols while focusing on details and theoretical aspects, do not translate directly into practical applications since a large number of samples need to be tested to validate them. Molecular imaging with mass spectrometry may offer ample possibilities in practical usage yet in the future rather than at present, because of extent of time necessary for analysis, workload of instruments, and poor image resolution in comparison to other methods [13-15].

Our work suggests an option of proteomic profiling of biological materials which focuses on obtaining spectra rich in proteins that may reveal the proteomic composition of tissue. Using multivariate statistical methods each complex spectrum can be shown as a single point in multidimensional space (as a single parameter), then analysed in respect of localization in this space. The single point in multidimensional space become a specific biomarker if its location does not match the control region. Even a slight change in tissue structure and the nature of pathology of any origin should be reflected in proteomic profile, provided that profiles richest in functional proteome of mass range of 3.5 - 20 kDa (without high abundant proteins - large proteins) are obtained. These profiles may correspond to a type of the pathological change (cancer, inflammation) as well as to normal profiles. The obtained profiles form something like a "proteomic fingerprint" and allow for classification of tested materials into appropriate groups using statistical methods. Tissue models have to be built for a known lung tissue structure (normal, changed: cancer, inflammation) using supervised classification methods. These methods indicate also signals typical for both normal and pathological tissue (table 1). Once appropriate models are calculated, any unknown lung tissue material can be measured and classified into defined groups (previously built models). The appearance of signals typical for a pathology results in classification of examined tissue into an appropriate model. This appears to be a simple protocol without complex, time consuming and expensive procedures which seems to be relevant considering a large number of samples needed for future models validation.

**Table 1:** Peak statistics - comparison of statistically important (p value of T-test/ANOVA < 0.05) peaks for data separation and recognition capability (biomarkers candidates).

Mass (Da)	Mean (Control)	Mean (Pathological)	P value T-Test/ANOVA	P value Wilcoxon/Kruskal-Wallis	Difference Average
8616.14	0.32	1.45	< 0.000001	0.00000847	< 0.000001

6228.05	1.55	4.07	< 0.000001	0.00152	< 0.000001

5759.35	1.54	2.65	< 0.000001	0.00017	0.000773

3588.09	1.99	4.82	< 0.000001	0.00017	< 0.000001

7596.85	0.81	2.11	< 0.000001	0.00154	< 0.000001

5201.26	1.93	3.87	< 0.000001	0.000366	< 0.000001

4156.39	2.08	4.87	< 0.000001	0.00017	0.0000021

9554.61	0.78	1.99	0.00000228	0.11	< 0.000001

12350.75	0.25	1.22	0.00000228	0.0000289	< 0.000001

3603.83	2.11	4.11	0.0000024	0.0162	< 0.000001

6723.40	20.93	8.96	0.00000228	0.00000855	0.000101

11297.49	0.45	0.97	0.00000228	0.00807	< 0.000001

10268.09	0.59	1.56	0.00000341	0.000314	< 0.000001

10525.25	0.3	1.19	0.00000695	0.00017	< 0.000001

5800.39	1.79	3.82	0.00000122	0.195	< 0.000001

5910.05	1.78	3.59	0.00000596	0.00375	< 0.000001

10403.69	0.47	1.43	0.000225	0.000331	< 0.000001

8297.66	0.95	1.62	0.000225	0.176	< 0.000001

4570.52	4.96	7.49	0.000296	0.106	< 0.000001

9440.24	0.71	1.08	0.000296	0.11	< 0.000001

6656.68	6.27	2.07	0.000608	0.00000986	< 0.000001

11325.26	0.67	1.09	0.00168	0.488	< 0.000001

9172.09	34.55	12.13	0.00171	0.00017	< 0.000001

7042.18	23.45	5.42	0.00252	0.0000902	< 0.000001

6020.90	5.33	2.28	0.00252	0.00017	< 0.000001

11185.69	0.75	1.19	0.00252	0.891	< 0.000001

4995.04	2.28	3.87	0.00409	0.162	< 0.000001

8456.35	1.25	2.82	0.00517	0.31	< 0.000001

9960.78	0.78	1.82	0.0059	0.211	< 0.000001

7283.97	39.59	7.2	0.00715	0.000151	< 0.000001

7249.02	2.69	1.77	0.00757	0.0042	0.00064

7173.62	4.21	7.46	0.00935	0.647	< 0.000001

5711.21	4.23	6.21	0.00935	0.441	< 0.000001

9378.33	1.82	1	0.00935	0.00238	< 0.000001

4048.24	4.11	7.16	0.0141	0.176	< 0.000001

5837.80	2.19	3.04	0.0197	0.423	< 0.000001

3907.34	41.93	14.71	0.021	0.00771	< 0.000001

10840.50	0.99	2.24	0.0231	0.149	0

6745.30	2.08	1.56	0.0392	0.0221	0.00817

## Methods

### Materials

Segments of specimens collected for an intraoperative consultation were examined. Only lung cancer suspected samples were taken into consideration. Diagnosis was made basing on segments prepared with a standard criostatic technique followed by revision on the basis of paraffin method from specimens stained with hematoxilin and eozine (HE) and complemented with immunohistochemical method. Pathological changes in tissues were classified according to The Word Health Organization classification. Types of pathology are listed in table 2.

**Table 2:** Types f pathological change diagnosed in examined material.

Diagnosis	Number of cases
Squamous cell carcinoma	25

Adenocarcinoma	27

Large cell carcinoma	4

Typical carcinoid	1

Large cell neuroendocrine carcinoma	3

Small cell carcinoma	2

Adenosquamous carcinoma	4

Sarconiatoid carcinoma	4

Bening tumors	2

Metastasis	2

Lymphoma	2

Ectopic tissue and tumor-like lesions	9

All the material for proteomic examination was collected from tissue fragments which did not show necrosis in volume in a macroscopic examination.

The control group (15 different cases) consisted of pathologically unchanged fragments of the lung tissue obtained from lungs removed during surgery on trauma cases. The material did not show any histological changes in histological examination that followed.

### Methods

Mass spectra were recorded in a linear mode of 3.1 - 20 kDa with a high resolution mass spectrometer of time of flight type (MALDI-TOF/TOF) Bruker version Autoflex III Smartbeam with 200 Hz laser technology. Sinapic acid (SA) of proteomic grade (Bruker) was used as a matrix. 400 mg of lung tissue was directly frozen at -80°C. Next, just before measurement, the material was defrozen, homogenized with 0.7 ml 0.9% NaCl solution and centrifuged at 13,000 rpm for 10 minutes. The obtained supernatant of 0.6 ml was treated with 50μl of 10% trifluoracetic acid (TFA) and centrifuged again and then it was transferred to Amicon Ultracel (Millipore) centrifugal filter of 3 kDa selectivity and centifuged at 13,000 rpm to decrease the volume to 50 μl. The concentrated solution containing proteins and peptides was desalted by twice repeated centrifugation in the same conditions with 450 μl of deionized water. Concentrated and desalted material of 10 μl was transferred to a conical tube and gently mixed with 20 μl of saturated SA (SA in water with 0.1% TFA and acetonitryl with 0.1% TFA at a ratio of 60:40). The obtained mixture was directly applied onto standard steel target and analyzed following solvent evaporation.

The instrument operated under control of FlexControl software (Bruker).

All the statistical calculations were done using ClinProtTols ver. 2.2 software (Bruker). Proteomic profiles were analyzed with principle component analysis algorithm (PCA) to observe differences in proteome profiles of particular groups. Supervised classification algorithms, Support Vector Machine (SVM), Genetic Algorithm (GA) and Supervised Neural Network (SNN) were used for classification of tissue signals into appropriate groups.

## Results

**Figure 1:**
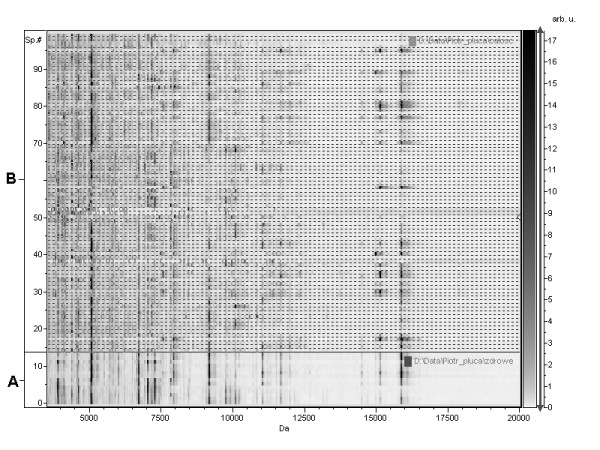
**MS spectra in gel view mode: a) control group, b) pathologically changed tissues**.

MS analysis in 100 (15 control, 85 pathological) tissue isolates was done. Results as combined spectra in gel view mode are shown in figure 1.

**Figure 2:**
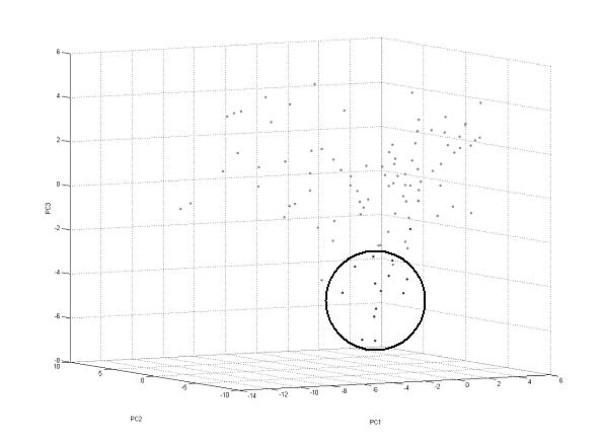
**The result of principle component analysis (PCA)**. Data distribution in three-dimensional space (PC1 - PC2- PC3). Points of control group were rounded.

All the spectra were analyzed with PCA algorithm and resulting data distribution in three-dimensional space view is presented in figure 2. Rounded points are the control group profiles and are marked for reasons of clarity.

Statistical analysis of all profiles for biomarker discovery revealed 39 masses of statistical importance (p < 0.005) which influenced division of investigated groups. Results are listed in table 1.

Proteome profiles were classified using three different supervised algorithms. Data for both (X1 - control, X2 - pathological) and recognition capability are listed in table 3.

**Table 3:** Classification results.

Algorithm	Validation			Recognition capability
		
	XVal	X1	X2	
Support Vector Machine (SVM)	95.8%	98.8%	92.9%	**100%**

Genetic Algorithm (GE)	95.3%	97.7%	92.9%	**100%**

Supervised Neural Network (SNN)	88.7%	98.8%	78.6%	**100%**

## Discussion

The proteomic investigation as a novel technique for biomarker discovery in disease process requires application of dedicated solutions including both advanced instruments and software. However, separation and concentration procedure are crucial for protein analysis [16]. Proteins of interest are a minority in comparison to a huge number of large structural proteins and other compounds which may negatively influence identification process especially in complex biological materials (blood, tissue) [17]. Moreover, mass spectroscopy with matrix associated laser desorption ionization, has its limitations: lack of highly abundant proteins in a sample, lack of salts, need for appropriate analytic concentrations, etc [18]. A number of protocols are described in literature for separation, concentration and desalting of proteins, however most of them are costly and time consuming [19-21]. We found that for peptides/proteins profiles purposes, considering the nature (composition - structure) of materials, it was enough to isolate only a limited range of proteins which could be presented as a single point in multidimensional space basic on statistical methods. A large number of mass signals recorded during measurement merge into one "signal" (a point located in multidimensional space) which can be treated as a specific biomarker. The obtained profiles (single points) become characteristic "fingerprints" for tissue composition. Any pathological changes in the examined material result in different localization in comparison to unchanged material what can be easy seen. Depending on the extent of pathological changes in tissue structure, proteins isolation procedure should include either more or fewer separation steps to obtain fractions which then can be successfully analyzed separately. In our approach we performed a simple isolation procedure based on precipitation of large proteins and then filtration using filters of cut-off level 3 kDa to concentrate and desalt peptides/proteins. This procedure allowed us to collect proteins in such a form that they can be measured directly in spectrometer without any expensive and time consuming procedures. As a detail examination of structure of particular peptides/proteins differentiating points distribution in multidimensional space was not our intent, we focused on reproducibility of this procedure. Our aim was to analyse profiles and compare them rather then to analyse a particular compound of which the profile was composed. Figure 1 presents spectra of all 100 samples, including control tissues (A) and pathologically changed (B) in gel view mode in order to combine all of them in one Figure 1. The data combination reveals that common signals may be noted in broad spectrum for both groups but pathological samples are even more complex in ranges between common bands. Signals in the same masses are most likely typical for the tissue origin (lung) and they reflect common to some extend structure for both types of the same tissues (pathological and normal). The multivariate statistical method enabled to distinguish normal samples from those diagnosed with cancer as well as those which indicate other pathological changes (table 2). As we were aware that signals typical for pathological tissue may overlap with signals coming from normal tissue which is present in material in every tumour growth in an unchanged form, we decided to examine a relatively large tissue segment of 400 mg, covering normal and pathological region. This facilitated testing specificity and sensitivity of the method with view to an early pathological change detection (in case the change cannot be seen macroscopically).

Recorded MS data typically comprise a large number of signals which makes it impossible to tell the difference between examined groups basing on direct raw MS spectra. However, the principle component analysis (PCA) algorithm enables to present complex spectrum in a single point located in multidimensional space. This location is strictly defined by sample composition, and the points representing spectra can be easily compared. The result of PCA analysis of all the samples shown in Figure 2 revealed different localization of normal and pathological samples. Moreover, pathological samples were more dispersed in space which could be explained by the fact that many different types of cancer were examined. We found 39 different masses (table 1) which are of statistical importance and discriminate all the data. The examination of compounds structure is a typical approach and has been widely described in references. We found that as much as 39 masses influence samples distribution in multidimensional space and all of them may be important for change identification. A typical approach based on a single, one-parametrical statistical investigation seems to be less efficient compared to multiparametrical methods especially in respect of sensitivity and selectivity. Also we attempted to point out that there was no need to precisely define (protein structure identification) each of 39 signals because all of them are strictly defined by the point located in multidimensional space (non - dimensional data x, y, z). Points obtained from PCA calculations can be then classified and compared using different classification algorithms (table 3). In our experiment the recognition capability of the tree most common models was 100% which may demonstrate a high specificity of the procedure.

## Conclusions

The proteomic investigation of tissue samples using advanced statistical methods enabled differentiation of lung tissue with cancer and unchanged tissue material even in an early stage of growth (low pathologcial to unchanged tissue ratio in sample). Following ist validation, this method, supporting commonly used histological techniques, may be considered an objective, precise and fast procedure in an early cancer diagnosis. Our protocol may be useful in procedures that need a large number of samples due to the elimination of time consuming steps. Multiparameter statistical methods may be helpful in elaboration of newly defined biomarkers for clinical applications expressed as many factors "in one point".

## Competing interests

The authors declare that they have no competing interests.

## Authors' contributions

PW collected and registered all the samples, performed histological examinations, participated in the design of the study, MS measurements and data analysis, participated in data analysis and interpretation. TJ conceived of the study, developed the methodology, carried out MS measurements, performed the data analysis and interpretation, drafted the manuscript. JA and TG collected segments of specimens and participated in clinical diagnosis. KB participated in design of the study and its coordination. All authors read and approved the final manuscript.

## References

[B1] Palmblad M Tiss A Cramer R Mass spectrometry in clinical proteomics - from the present to the future Proteomics Clin Appl 2009 3 6 17 10.1002/prca.200800090 21136932

[B2] Munro NP Cairns DA Clarke P Rogers M Stanley AJ Barrett JH Harnden P Thompson D Eardley I Banks RE Knowles MA Urinary biomarker profiling in transitional cell carcinoma Int J Cancer 2006 119 2642 2650 10.1002/ijc.22238 16991122

[B3] Coombes KR Morris JS Hu J Edmonson SR Baggerly KA Serum proteomics profiling - a young technology begins to mature Nature Biotechnol 2005 23 291 292 10.1038/nbt0305-291 15765078

[B4] Check E Proteomics and cancer: running before we can walk Nature 2004 429 496 497 10.1038/429496a 15175721

[B5] Zhang X Leung SM Morris CR Shigenaga MK Evaluation of a novel, integrated approach using functionalized magnetic beads, bench-top MALDI-TOF-MS with prestructured sample supports, and pattern recognition software for profiling potential biomarkers in human plasma J Biomol Technol 2004 15 167 175 PMC2291685 15331582

[B6] Li J Zhang Z Rosenzweig J Wang YY Chan DW Proteomics and bioinformatics approaches for identification of serum biomarkers to detect breast cancer Clin Chem 2002 48 1296 1304 12142387

[B7] Petricoin EF Ardekani AM Hitt BA Levine PJ Fusaro VA Steinberg SM Mills GB Simone C Fishman DA Kohn EC Liotta LA Use of proteomic patterns in serum to identify ovarian cancer Lancet 2002 359 572 10.1016/S0140-6736(02)07746-2 11867112

[B8] Conrads TP Zhou M Petricoin EF Liotta LA Veenstra TD Cancer diagnosis using proteomic patterns Expert Rev Mol Diagn 2003 3 411 10.1586/14737159.3.4.411 12877381

[B9] Aebersold R Goodlett DR Mass spectrometry in proteomics Chem Rev 2001 101 269 295 10.1021/cr990076h 11712248

[B10] Stoeckli M Staab D Schweitzer A Compound and metabolite distribution measured by MALDI mass spectrometric imaging in whole-body tissue sections Int J Mass Spectrom 2007 260 195 202 10.1016/j.ijms.2006.10.007

[B11] Andersson M Groseclose MR Deutch AY Caprioli RM Imaging mass spectrometry of proteins and peptides: 3 D volume reconstruction Nat Methods 2008 5 101 108 10.1038/nmeth1145 18165806

[B12] Caldwell RL Caprioli RM Tissue profiling by mass spectrometry: A review of methodology and applications Mol Cell Proteomics 2005 4 394 401 10.1074/mcp.R500006-MCP200 15677390

[B13] Chaurand P Norris JL Cornett DS Mobley JA Caprioli RM New developments in profiling and imaging of proteins from tissue sections by MALDI mass spectrometry J Proteome Res 2006 5 2889 2900 10.1021/pr060346u 17081040

[B14] Cornett DS Reyzer ML Chaurand P Caprioli RM MALDI imaging mass spectrometry: Molecular snapshots of biochemical systems Na Methods 2007 4 828 833 10.1038/nmeth1094 17901873

[B15] Khatib-Shahidi S Andersson M Herman JL Gillespie TA Caprioli RM Direct molecular analysis of whole-body animal tissue sections by imaging MALDI mass spectrometry Anal Chem 2006 78 6448 6456 10.1021/ac060788p 16970320

[B16] Rifai N Gillette MA Carr SA Protein biomarker discovery and validation: The long and uncertain path to clinical utility Nat Biotechnol 2006 24 971 983 10.1038/nbt1235 16900146

[B17] Duncan R McConkey EH How many proteins are there in a typical mammalian cell? Clin Chem 1982 28 749 755 7074868

[B18] Diamandis EP Mass spectrometry as a diagnostic and a cancer biomarker discovery tool: opportunities and potential limitations Mol Cell Proteomics 2004 3 367 378 10.1074/mcp.R400007-MCP200 14990683

[B19] Omenn GS Strategies for plasma proteomic profiling of cancers Proteomics 2006 6 5662 5673 10.1002/pmic.200600331 16991194

[B20] Rifai N Gillette MA Carr SA Protein biomarker discovery and validation: The long and uncertain path to clinical utility Nat Biotechnol 2006 24 971 983 10.1038/nbt1235 16900146

[B21] Görg A Weiss W Dunn MJ Current two-dimensional electrophoresis technology for proteomics Proteomics 2004 4 3665 3685 15543535 10.1002/pmic.200401031

